# An Optical Method for the Rapid Measurement of Corrugated Plate Depth Based on Line Laser Sensor

**DOI:** 10.3390/s26082446

**Published:** 2026-04-16

**Authors:** Jie Chen, Xudong Mao, Xin Li, Qiuying Zhou, Changhui Huang, Chengxing Wu

**Affiliations:** 1 Institute of Special Equipment Inspection and Research, Jiangxi General Institute of Testing and Certification, Nanchang 330029, China; ncuchenjie@126.com (J.C.); lixin1924@nuaa.edu.cn (X.L.); zqy8212979@126.com (Q.Z.); jxgj106@163.com (C.H.); 2 Institute of Metrology, Jiangxi General Institute of Testing and Certification, Nanchang 330029, China; maoxudong_ustb@163.com; 3 Huazhong Institute of Electro-Optics, Wuhan 430223, China

**Keywords:** corrugated plate, corrugation depth, line laser sensor, data condensation, b-spline fitting

## Abstract

**Highlights:**

**What are the main findings?**

- Introduced a non-contact optical sensing method using a line laser and precision linear guide for rapid surface profile acquisition of corrugated plates.
- Developed a data-driven feature extraction approach combining multi-stage profile reduction and coarse-to-fine B-spline peak–valley detection, achieving accurate depth measurement.

**What are the implications of the main findings?**

- Provides a scalable framework for high-resolution, automated surface morphology measurement applicable to structured or repetitive geometries.
- Demonstrates the effectiveness of combining optical sensing with advanced data processing for reliable and efficient geometric parameter extraction in remote sensing applications.

**Abstract:**

This paper presents a non-contact depth detection method for corrugated heat exchanger plates, aiming to improve measurement efficiency and accuracy. The system integrates a line laser sensor with a precision linear guide rail, enabling continuous acquisition of high-resolution 2D surface profiles as the sensor moves along the plate. To reduce data redundancy while preserving geometric features, a multi-stage data reduction strategy is proposed. This strategy combines the angle–chord height criterion with spline-based filtering to identify key regions of curvature and eliminate unnecessary point cloud data. For depth extraction, a two-stage feature recognition algorithm is designed. First, a coarse analysis locates candidate peaks and valleys by identifying local extrema in the reduced 2D data. Then, a fine detection process is applied: local B-spline fitting is performed near each candidate point, and a binary search algorithm is used to accurately determine the spline extrema. By computing the vertical distance between precisely located peaks and valleys, the system rapidly extracts the corrugation depth parameters. This method achieves a high balance between speed and precision, offering a practical and reliable solution for automated surface morphology inspection in heat exchanger manufacturing.

## 1. Introduction

The plate heat exchanger, a key component in heat exchange systems, is made of metal corrugated plates with complex geometries. These plates form narrow rectangular channels that enable efficient heat transfer [1,2]. The uniformity of the corrugation depth significantly influences thermal performance by affecting turbulence generation and heat exchange efficiency [3,4]. Therefore, precise and efficient measurement of corrugation depth is essential for optimizing energy utilization and ensuring product quality [5]. While simple, these methods are inherently inefficient, prone to operator-dependent errors, and susceptible to missed measurements, making them unsuitable for high-throughput industrial inspection scenarios. In contrast, the rapid development of sensor and imaging technologies has led to the widespread adoption of non-contact inspection strategies in manufacturing quality control [6,7]. These approaches integrate advanced sensing hardware with sophisticated data acquisition and processing algorithms to enable high-precision, high-efficiency automated inspection solutions [8].

Geometric data acquisition methods for industrial measurement applications are generally categorized into several types, among which ultrasonic [9] and optical [10] techniques are the most widely adopted due to their versatility and adaptability across a range of inspection scenarios. However, the effectiveness of ultrasonic testing is highly dependent on the surface condition of the target object and requires appropriate coupling materials, which limits its applicability to components with highly curved or irregular surfaces [11]. In contrast, non-contact optical methods have attracted growing attention due to their high precision, fast acquisition speed, and seamless integration into automated workflows. These techniques offer robust, non-destructive, and cost-effective solutions for measuring components with complex geometries [12].

Non-contact optical methods are typically classified into three categories based on the type of projected light source: point laser, line laser, and structured light using area-array projection [13]. Each offers different trade-offs in terms of spatial resolution, measurement coverage, and acquisition efficiency. Extensive applied research has been conducted globally to optimize these technologies for various industrial scenarios [14,15]. Among these, point laser sensors represent one of the earliest and most widely adopted solutions for high-precision measurement tasks. For example, Cardoso et al. [16] employed that to evaluate deformation in agricultural machinery components, demonstrating its feasibility for capturing displacement behaviors with high accuracy by comparing experimental results against finite element simulations. In another study, Wu et al. [17,18] proposed two point-laser-based inspection systems tailored for detecting thinning defects in corrugated plates. By designing different sampling trajectories, they achieved high-precision measurements aligned with specific defect characteristics. However, due to their single-point sampling nature, point laser sensors require dense scanning and high-precision motion platforms, which limits their speed and scalability. As a result, these systems often fall short of meeting the growing demand for high-efficiency, low-cost industrial inspection [19].

To address these limitations, research efforts have increasingly focused on advanced optical measurement strategies that combine high precision with high throughput. In this context, structured light and line laser technologies have emerged as two of the most promising non-contact solutions [20]. Notably, structured light-based 3D measurement systems are well-regarded for their ability to rapidly acquire dense surface data across large areas without requiring mechanical scanning [21]. This capability makes them especially suitable for high-end manufacturing applications involving complex geometries and stringent accuracy requirements [22]. For example, Zhang et al. [23,24] achieved efficient 3D measurement of automotive body panels by employing techniques such as multiple exposure modulation and optimized viewpoint planning, tailored to the complexity of the surfaces. Wu et al. [25] enhanced robotic sorting capabilities in cluttered, stacked environments by developing part-type identification strategies based on point cloud feature analysis. Xiao et al. [26] implemented multi-view point cloud stitching techniques to enable accurate large-scale object measurement.

Despite their advantages, structured light systems are inherently sensitive to surface reflectivity and geometric complexity, particularly when inspecting highly reflective or irregularly shaped objects [27]. These limitations constrain their applicability in industrial environments where surface properties vary or are difficult to control. In contrast, line laser technology based on single-line projection offers a robust alternative for high-precision, high-efficiency measurement of complex components [28,29]. A key advantage of line laser systems is their active exposure suppression capability, which enhances measurement stability under varying surface conditions [30,31,32]. For instance, Huang et al. [33] developed a simplified, high-precision calibration method for laser scanning systems, enabling accurate 3D reconstruction of plaster objects with intricate geometries and metallic components with uneven reflectivity. Wang et al. [34] proposed a feature-matching algorithm based on the covariance matrix of local leading-edge features to address challenges in turbine blade profile measurement. Usamentiaga et al. [35] developed a flatness measurement system for rails employing a dual triangulation configuration, effectively addressing critical issues such as sensor calibration, data registration, and flatness characterization. Additionally, Yu et al. [36] developed an optical inspection approach integrating line laser sensors and vision cameras, enabling efficient and accurate on-line measurement of small modulus gears.

While line laser sensors effectively balance measurement accuracy and operational efficiency, they also produce substantial volumes of point cloud data during scanning. Direct computation of critical dimensions from such large-scale datasets is computationally intensive and susceptible to error propagation [37,38]. As a result, point cloud data reduction has become a crucial preprocessing step. Among current methods, geometry-based reduction techniques are most widely adopted, typically segmenting point clouds into feature and non-feature regions by analyzing geometric attributes such as curvature, normal vector variation, and surface continuity [39]. For instance, Shamshe et al. [40] proposed a reduction strategy based on edge extraction, effectively preserving boundary features while removing redundant points. Similarly, Gao et al. [41] developed an algorithm that employs angle–coordinate threshold correlation analysis to guide data reduction according to geometric feature thresholds. Building on these approaches, Pan et al. [42] introduced a point cloud processing method for complex weld seams, which aligns multi-frame data through coordinate transformations and extracts weld features using the RANSAC algorithm. However, a common limitation of these approaches is the lack of a systematic validation mechanism to evaluate the quality of the reduced data. Without such verification, critical geometric information may be lost, potentially undermining the accuracy of subsequent measurements.

To overcome the limitations of existing approaches, this study proposes a comprehensive algorithmic framework that not only facilitates efficient and intelligent point cloud data reduction, but also incorporates robust quality evaluation metrics to ensure measurement accuracy and reliability in industrial inspection workflows. Unlike traditional reduction strategies that emphasize multi-feature geometric analysis, the focus here is on developing an end-to-end methodology for mechanical part inspection—particularly with respect to specification compliance. A targeted data processing algorithm is implemented to ensure that measurement results maintain a suitable uncertainty-to-tolerance ratio. By extracting key parameters and quality indicators from the acquired data, an automated inspection strategy is formulated to evaluate components against predefined specifications. The main contributions of this study are as follows:

(1) A robust data reduction strategy that incorporates angle and chord height constraints is proposed;

(2) A coarse-to-fine feature recognition algorithm is developed to identify a representative set of feature points;

(3) A specification-oriented optical inspection approach is established for the preliminary analysis of complex components.

The remainder of this paper is organized as follows. Section 2 introduces the measurement system based on a line laser sensor. Section 3 presents the data preprocessing strategy, including the proposed data reduction method. Section 4 outlines the procedure for identifying corrugation height parameters. Section 5 reports the experimental results, and Section 6 concludes the study.

## 2. Measurement System

**Figure 1:**
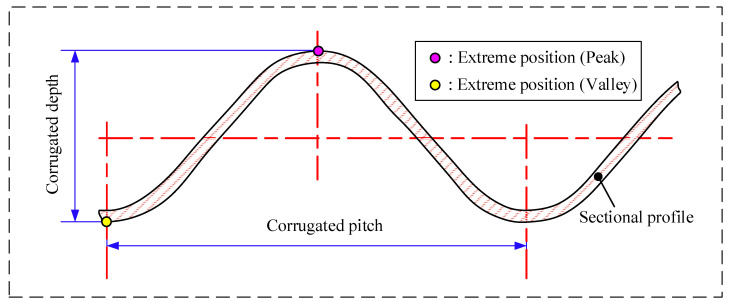
Geometric definition of corrugation depth.

This study focuses on the complex morphology of the chevron-type corrugated plate, with particular emphasis on its corrugated depth h. Corrugated depth is usually defined as the difference between the highest and lowest positions within a single corrugated pitch λ [3] (see Figure 1). The variation in corrugated depth directly impacts the plate’s load-bearing capacity. It also influences fluid flow behavior within the channel, which in turn affects the heat transfer performance of the heat exchanger. Therefore, a high-precision measurement of the corrugated depth is crucial for optimizing the structure and evaluating the heat exchanger’s performance.

**Figure 2:**
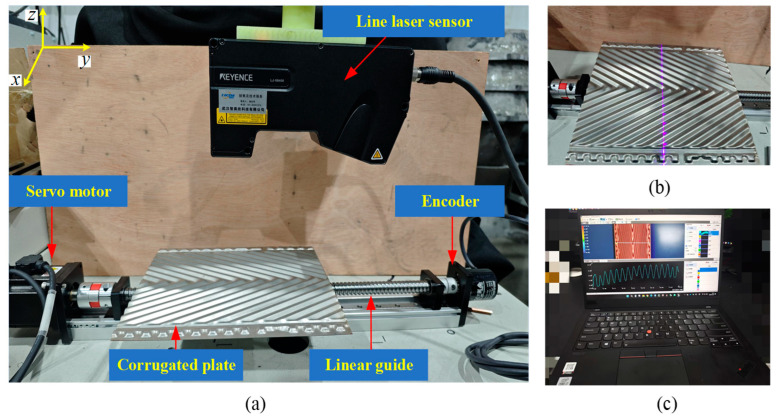
Measurement system with line laser scanning technology: (**a**) overall structure design; (**b**) measurement schematic; (**c**) data acquisition software (Keyence LK-Navigator2 (Keyence Corporation, Osaka, Japan)).

To enable the high-precision detection of corrugated depth, the key lies in the accurate identification of the extreme points (i.e., the highest and lowest points) of the plate’s cross-sectional profile. In this study, an efficient measurement system based on line laser scanning technology has been developed (as shown in Figure 2) to precisely capture the complex three-dimensional morphology of the corrugated plate. The system is composed of a LJ-X8400 line laser sensor (Keyence Corporation, Osaka, Japan; detailed performance parameters listed in Table 1), a high-precision linear guide, a Huichuan servo motor (Inovance Technology Co., Ltd., Shenzhen, China), and an Omron E6B2 encoder (Omron Corporation, Kyoto, Japan).

The system employs a synchronized sampling mechanism triggered by the encoder to ensure spatial consistency during data acquisition. The servo motor drives the linear guide to move in the horizontal direction with an accuracy of 0.01 mm, while the E6B2 encoder outputs high-precision signals with 2000 pulses per revolution to provide accurate position feedback. By setting the line laser sensor to operate in encoder-triggered sampling mode, data is synchronized with the trigger signal from the encoder. This ensures data is collected exactly when the signal is issued. The synchronized sampling mechanism effectively mitigates data deviation caused by motion errors during the sampling process, significantly enhancing the accuracy and stability of the measurement data. Consequently, this approach provides reliable technical support for the efficient and precise measurement of the corrugated plate.

## 3. Data Preprocessing with Data Reduction

**Figure 3:**
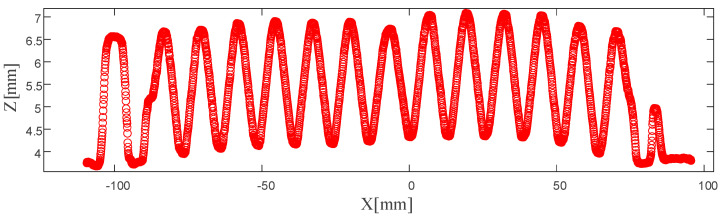
Original measurement data for the corrugated plate cross-section.

As shown in Figure 3, the collected measurement data of the corrugated plate cross-section reveal its surface morphology characteristics. The LJ-X8400 laser line sensor used in this study is capable of capturing up to 3200 data points in a single scan, resulting in a large-scale dataset. Meanwhile, due to the reflective characteristics of the corrugated plate surface and the inherent measurement mechanism of line laser scanning, the acquired data may contain local fluctuations, intensity-induced deviations, and small outliers. These uncertainties arise from variations in surface reflectivity, signal noise, and point localization errors during the reconstruction of the profile. Although such deviations may not be prominently visible in the global profile, they can introduce disturbances in local geometric features. If depth extraction is performed directly on the raw measurement data, these noise-induced variations may affect the robustness of feature identification, particularly in the detection of extrema and geometric transitions. In addition, processing dense raw datasets without any reduction or preprocessing would lead to increased computational burden and reduced efficiency. Therefore, to improve both the stability of feature extraction and the efficiency of subsequent analysis, it is necessary to perform data preprocessing prior to depth evaluation. This step helps suppress the influence of measurement uncertainty and provides a more reliable representation of the underlying surface profile. At the same time, key curvature features of the corrugated plate are preserved, thereby improving the reliability of subsequent depth extraction.

**Figure 4:**
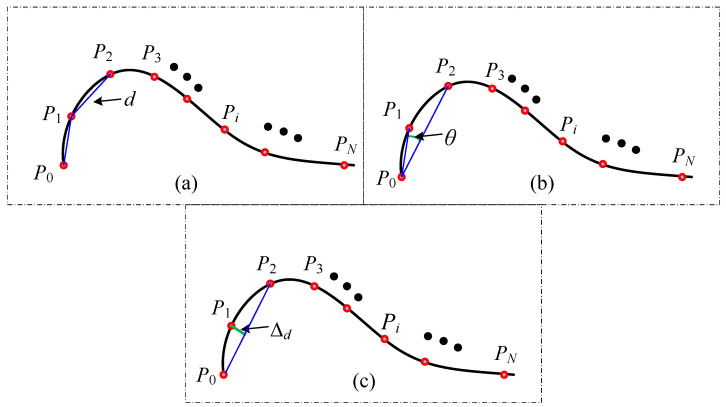
Common data reduction methods: (**a**) minimum distance method, (**b**) angle deviation method, (**c**) chord deviation method.

**Figure 5:**
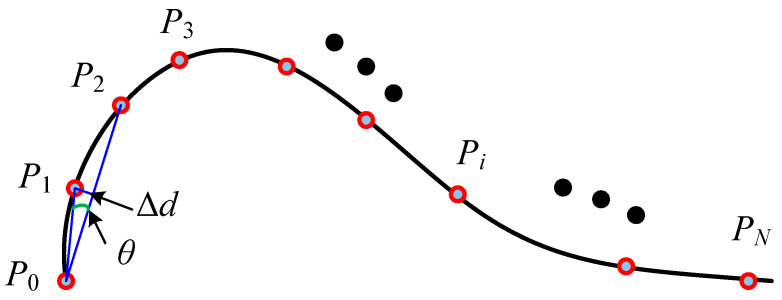
An improved data reduction method with the angle-chord deviation.

As shown in Figure 4, commonly data reduction methods include the minimum distance method, the angle deviation method, and the chord deviation method [39]. The minimum distance method reduces data by constraining the minimum allowable distance between these consecutive points. If the distance between two adjacent points satisfies, the redundant point is removed to optimize data density. The angle deviation method filters data based on the angular variation between vectors formed by consecutive points. With the angular change, excessive points are eliminated. Similarly, the chord deviation method removes redundant points by imposing a constraint on the chord height difference threshold. If the condition is met, points within the chord length are discarded to refine the dataset. These methods primarily rely on curvature-based feature evaluation for data reduction. However, when applied individually, they may result in suboptimal data filtering, potentially affecting accuracy and completeness. To achieve a more efficient and reasonable data reduction process, an improved angle-chord deviation method is developed in this study for preprocessing line laser scanning data. The key steps of this approach are illustrated in Figure 5.

**Step 1:** The tolerance values for the angle $\theta_{min}$ and the chord height ${∆d}_{min}$ are defined.

**Step 2:** Three consecutive data points, *P*_0_, *P*_1_ and *P*_2_, are selected starting from the initial measurement data. The angle θ between vectors $\vec{P_{0}P_{1}}$ and $\vec{P_{0}P_{2}}$, as well as the chord height, is then computed.

(1) $$\left\{\begin{array}{l} \theta =arccos(\frac{\vec{P_{0}P_{1}}\cdot \vec{P_{0}P_{2}}}{\left\|\vec{P_{0}P_{1}}\right\|\left\|\vec{P_{0}P_{2}}\right\|})\\ ∆d=\left\|\vec{P_{0}P_{1}}\right\|sin(\theta ) \end{array}\right.$$

**Step 3:** If the angle θ is smaller than the predefined threshold $\theta_{min}$ and the distance variation ∆d is less than the threshold ${∆d}_{min}$, point *P*_1_ is identified as a redundant data point and should be removed. Subsequently, the point cloud sequence is updated by assigning *P*_2_ as the new *P*_1_ and *P*_3_ as the new *P*_2_. If *P*_3_ does not exist, the data reduction process is considered complete; otherwise, the procedure returns to **Step 2** for further iterations.

**Step 4:** If neither of the conditions $\theta <\theta_{min}$ and $∆d<{∆d}_{min}$ is satisfied simultaneously, the point cloud sequence is updated by assigning *P*_1_ as the new *P*_0_ and *P*_2_ as the new *P*_1_, while the next point after *P*_2_ is selected as the new *P*_3_. If *P*_3_ does not exist, the point cloud simplification process is considered complete; otherwise, the procedure returns to **Step 2** for further iterations.

**Figure 6:**
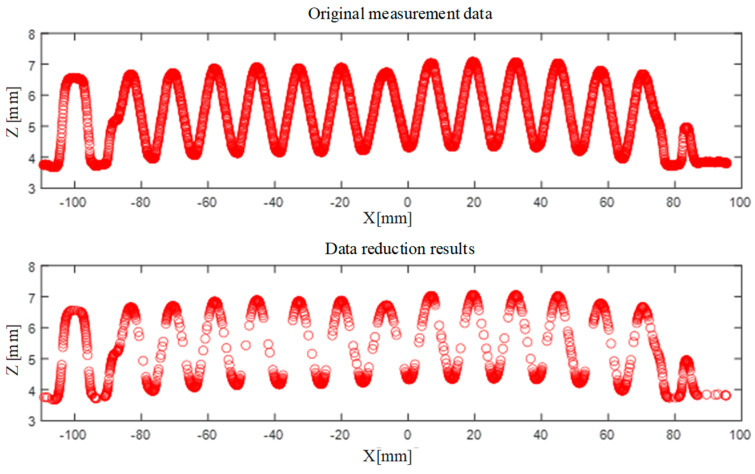
Reduction results for the measurement data of corrugated plate cross-section.

As shown in Figure 6, the raw data acquired by the LJ-X8400 line laser sensor camera consists of 3200 data points. By setting threshold values for allowable angle and chord height, the data was processed and reduced to 1086 points. From the figure, it can be observed that the employed data reduction algorithm effectively preserves the characteristic features of the original curve while significantly compressing the data size. This ensures measurement accuracy while enhancing data processing efficiency.

## 4. Identification of Corrugated Height

**Figure 7:**
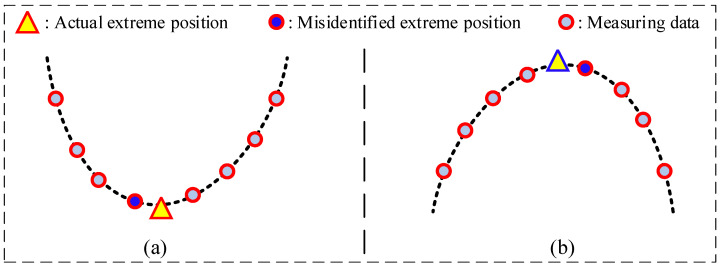
Misidentification of extreme value positions: (**a**) trough; (**b**) wave peak.

After the measurement data of the corrugated plate has been reduced, to accurately extract the peak and valley feature points for depth calculation, it is necessary to further identify the extreme values of the cross-sectional profile data. However, when extreme value points are directly selected from the discrete measurement data, calculation errors may arise, as shown in Figure 7, due to these measurement data not aligning precisely with the peaks or valleys. To address this issue, considering that the design profile of the corrugated plate is a smooth curve, a curve fitting strategy can be applied to optimize such procedure. Based on that, the extreme points for the sectional profile of a corrugated plate can be accurately extracted, thereby improving the precision and stability of the depth calculation.

When fitting the curve to the global point cloud data, challenges such as low efficiency and poor precision may occur due to the large data volume. To address this, a multi-step optimizing-search algorithm combining fine and rough adjustment is proposed. Initially, key points are extracted within the measurement data. Once the key points are identified, local B-spline fitting is applied to the surrounding data. Finally, a local fitting curve is generated. This allows for the accurate extraction of extreme points at the peaks and valleys of the corrugated plate. This method ensures precise results for calculating corrugated depth.

### 4.1. Procedure I: Key Points Extraction

For the pre-processing data $P=\{P_{0},P_{1},\cdots P_{i},\cdots P_{N}\}$ for one cross-section plate shown in Figure 6, where *N* represents the total number of data, Firstly, a first-order difference is applied to the data ***P***, and the results $dP=\{{dP}_{0},{dP}_{1},\cdots {dP}_{i},\cdots {dP}_{N}\}$ are symbolized to compute as follows:

(2) $$\left\{\begin{array}{l} {dP}_{i}=sign\left(P_{i+1}-P_{i}\right),\;i=1,\cdots ,N-1\\ {dP}_{N}=sign(\frac{P\left(N-2\right)+2\times P\left(N-1\right)-3\times P\left(N\right)}{6}) \end{array}\right.$$

where $sign\left(x\right)=\left\{\begin{array}{l} 1\;x>0\\ 0\;x=0\\ -1\;x<0 \end{array}\right.$ is a sign function.

Furthermore, by performing a differential operation on dP, the following result is obtained:

(3) $$\left\{\begin{array}{l} {d^{2}P}_{i}=sign\left({dP}_{i+1}-{dP}_{i}\right),\;i=1,\cdots ,N-1\\ {d^{2}P}_{N}=sign(\frac{dP\left(N-2\right)+2\times dP\left(N-1\right)-3\times dP\left(N\right)}{6}) \end{array}\right.$$

**Figure 8:**
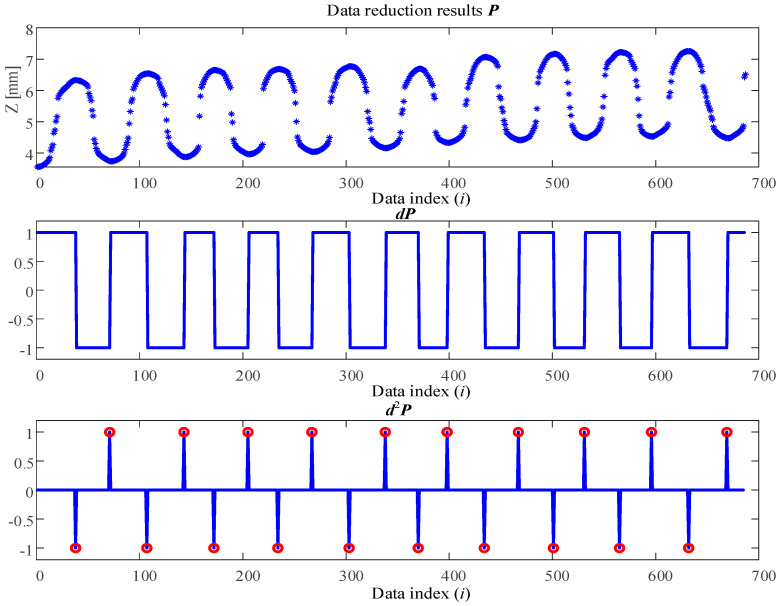
Key point extraction for the reduction data of corrugated plate cross-section.

The differential results are illustrated in Figure 8. It should be emphasized that the *X*-axis in Figure 8 does not represent the original physical measurement length. Instead, it denotes the sequential indices of the sampled key points obtained after the preprocessing and data reduction procedure. Due to the re-indexing of the reduced dataset, the numerical range of the *X*-axis appears larger than the actual measurement length reported in Table 1 (i.e., 180 mm). Therefore, the *X*-axis in Figure 8 should be interpreted as the ordering of the extracted points rather than a physical distance scale. As shown in the figure, the data points satisfying the condition $abs\left({d^{2}P}_{i}\right)=1$, which are highlighted with circles, are identified as feature key points corresponding to wave peaks and valleys. The total number of key points extracted from a single cross-section is denoted as M.

### 4.2. Procedure II: Sectional Profile Reconstruction

After completing the extraction of key points from the reduction data, directly treating these points as the true peak and valley positions of the corrugated plate may lead to errors, as illustrated in Figure 7. While a global cross-section fitting reconstruction approach could provide a more comprehensive representation, it requires significant computational resources and may suffer from accuracy loss. To overcome these issues, a local B-spline fitting method is used. This improves the precision of feature point extraction for the corrugated plate.

**Figure 9:**
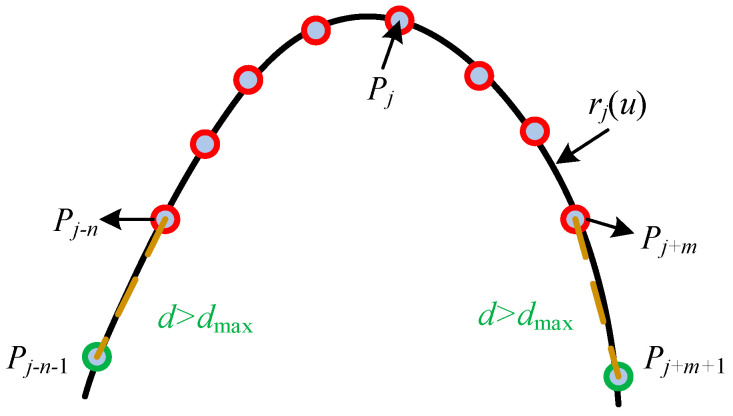
Schematic diagram of fitting data search at these key point positions.

As shown in Figure 9, the selection of fitting points for the j-th peak or valley feature key point $P_{j}$ is performed based on a distance criterion. Specifically, starting from $P_{j}$, neighboring points are iteratively indexed in both the left and right directions while adhering to predefined constraints. A maximum search distance $d_{max}$ and a maximum number $n_{max}$ of search points are respectivelly set as $d_{max}=1.5\;mm$ and $n_{max}$ = 5. The number of data points retrieved on the left is denoted as n, while that on the right is m. Ultimately, this search process yields an initial set of k=m+n+1 points, which is subsequently utilized for B-spline curve fitting. By employing this method, the accuracy and robustness of extremal point detection are significantly enhanced.

To achieve precise extraction of points at these positions of the corrugated plate’s peaks and valleys, a cubic B-spline fitting method is employed for data interpolation. During the fitting process, continuity of the first derivative at both endpoints is enforced to ensure the smoothness of the curve. Consequently, the resulting smooth spline function is expressed as $r_{j}(u)$:

(4) $$r_{j}\left(u\right)=\sum_{i=0}^{M}B_{i,k-1}(u)C_{i}$$

In this context, $B_{i,k-1}(u)$ represents the B-spline basis function, while $C_{i}$ denotes the control points. The total number of control points satisfies M=k+p, where the spline degree is set to p=3. The centripetal method is used for parameter computation. It determines the parameter values for each fitted point. This ensures a well-distributed parameterization. It also improves the stability of the spline fitting process.

(5) $$\left\{\begin{array}{l} u_{1}=0,u_{k}=1\\ d=\sum_{i=2}^{k}\left|\vec{P_{i-1}P_{i}}\right|\\ u_{i}=u_{i-1}+\frac{\left|\vec{P_{i-1}P_{i}}\right|}{d} \end{array}\right.,i=2,\cdots ,k-1$$

where *u* represents the B-spline parameter corresponding to the fitted point.

To ensure the smoothness of the B-spline curve with respect to these fitting data, specific constraints in Equation (6) are imposed on the spline formulation.

(6) $$\left\{\begin{array}{l} r\left(u_{i}\right)=P_{i}^{\prime }\\ r^{\prime }\left(0\right)=\vec{P_{j-n-1}P_{j-n}}\\ r^{\prime }\left(1\right)=\vec{P_{j+m}P_{j+m+1}} \end{array}\right.$$

**Figure 10:**
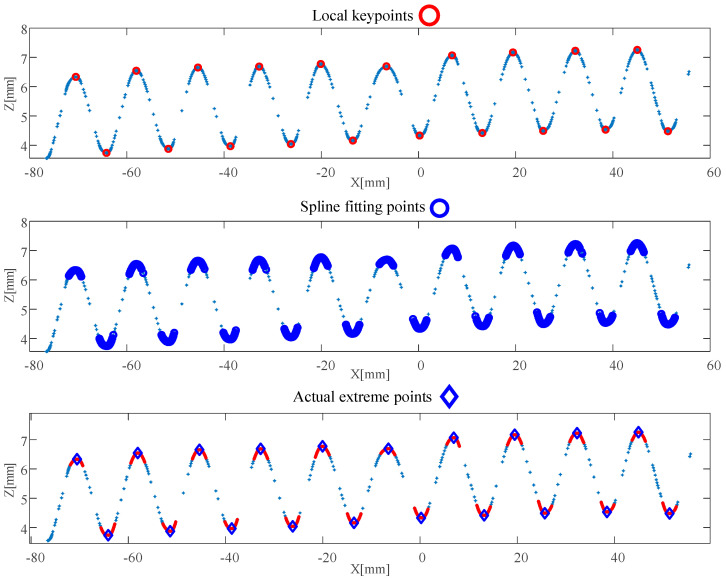
Extraction results for the sectional profile extremum values.

By substituting these constraints into Equation (4), the control points C can be determined. Based on this, a local B-spline curve $r_{j}(u)$ is subsequently constructed. Since the constructed spline curve contains only a single extremum, its derivative is computed to identify the point $\bar{P_{jj}}(\bar{x_{jj}},\bar{y_{jj}})(jj=1\cdots M)$ that satisfies condition $r^{\prime }\left(\bar{u}\right)=0$. This point is regarded as the true peak or valley feature corresponding to the local characteristic point $P_{j}$. The extraction results are presented in Figure 10.

After identifying the extreme points of the peaks and valleys, the final depth of the corrugated plate can be determined by calculating the distances between adjacent peaks and valleys. This process relies on the spatial distribution of the feature points. Distance measurement techniques are then used to ensure accuracy and reliability in evaluating the depth.

## 5. Experimental Results and Discussion

Based on the data analysis strategy designed in Section 3 and Section 4, the effectiveness of the developed measurement method for the identification of corrugated plate depth was experimentally tested. By applying the established measurement system in Section 2, the test was conducted using a chevron-patterned corrugated plate made of 304 stainless steel, with dimensions of 170×205 mm. The design dimension for the corrugated depth in the central area of the plate is 2.3~2.5 mm. To balance precision and efficiency, the movement speed of the linear guide rail was matched with the sampling response speed of the laser sensor triggered by the encoder. Specifically, the linear guide rail was set to move at a speed of 5 mm/s, while the encoder was configured to trigger the laser sensor to collect data every 10 encoder pulses. Given that the lead of the linear guide rail is 5 mm and the encoder provides 2000 pulses per revolution, the signal sampling was effectively carried out at intervals of 0.025 mm along the direction of rail movement.

**Figure 11:**
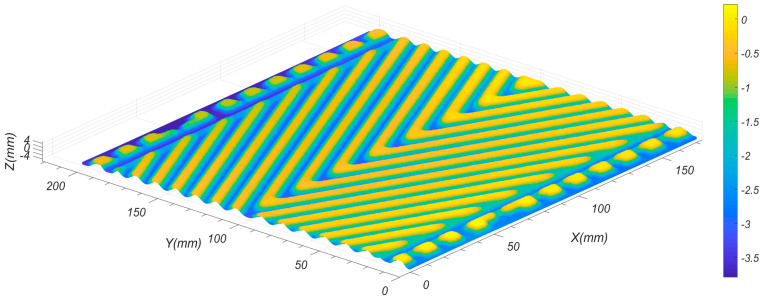
Registration and reduction results for the measurement data of test object.

**Figure 12:**
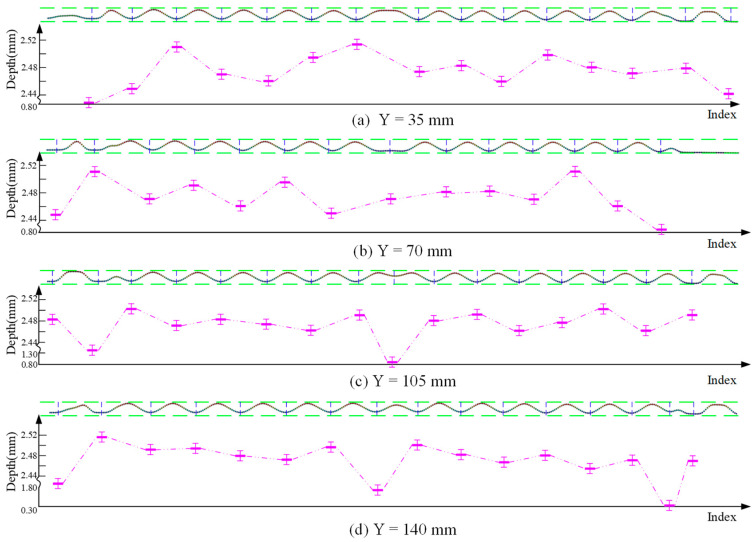
The identification results for corrugated plate depth at Y=35,70,105,140 mm.

By performing the reduction and registration process to the original measurement data, the complete surface data of the test plate can be obtained. The corresponding result, shown in Figure 11, was generated using MATLAB R2017a. It can be clearly observed that redundancy in the measurement data has been eliminated, while key morphological features have been preserved for further analysis. Figure 12 illustrates the calculated results of corrugated depth for several cross-sections at Y=35,70,105,140 mm. In Figure 12, the horizontal axis labeled as “index” refers to the measurement point index, which denotes the sequential order of sampled data points along the scanning direction rather than a physical spatial coordinate. It can be observed that, with the exception of certain localized areas influenced by geometric constraints, the calculated corrugated depths $h_{c}$ are mostly within the range of 2.4 mm to 2.5 mm. This range is largely consistent with the metal flow patterns observed during the stamping process of the corrugated plate [43].

**Figure 13:**
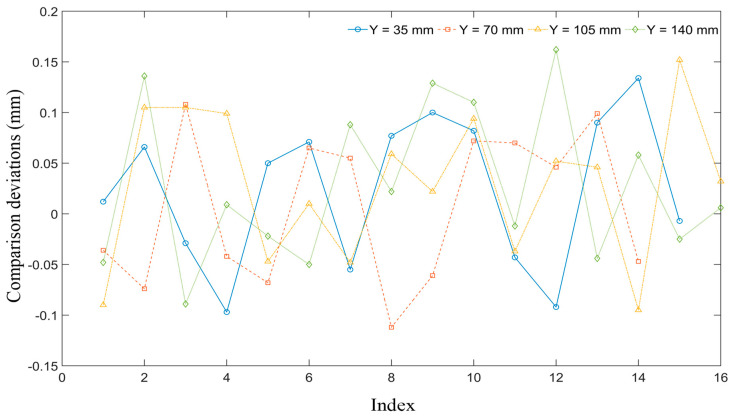
Comparison results between the calculated and CMM measurement results.

The results of the proposed method were compared with those obtained using a coordinate measuring machine (CMM). The results $h_{0}$ for the CMM are defined as the benchmark. Figure 13 presents the comparison deviation of corrugation depth within local regions along selected cross-sections (Y=35,70,105,140 mm). The horizontal axis denotes the index of the local regions, while the vertical axis represents the comparison deviation ∆e (∆e = $h_{c}-h_{0}$). The majority of the alignment deviations are positive, primarily due to factors like surface reflection and scanning angle occlusion during the line laser probe scanning process. These factors caused distortions or gaps in the point cloud data, leading to overestimation of some measurement values. In contrast, the few negative deviations observed may be attributed to local deformations caused by the probe’s contact force during the CMM measurement process, especially with thin-walled components, resulting in underestimated values.

However, the maximum relative alignment deviation remains below 17%, and this deviation is evaluated with respect to the reference values obtained from the CMM measurements. In this study, the deviation is defined as the relative difference between the estimated results and the corresponding CMM reference values. While this level of deviation indicates a noticeable discrepancy, it is still considered reasonable for corrugated plate depth measurement, given the inherent uncertainties associated with both the measurement process and the reference data, as well as the typical tolerance levels encountered in industrial applications. Overall, the results suggest that the proposed method provides a satisfactory level of accuracy, thereby supporting its validity and practical applicability. In addition, the proposed method substantially reduces the measurement time compared with conventional coordinate measuring machine (CMM)-based approaches, achieving an order-of-magnitude improvement. This indicates its potential efficiency advantages in practical inspection scenarios.

To evaluate the effectiveness of the proposed preprocessing strategy, a comparative experiment was conducted using a uniform subsampling (downsampling) method as a baseline. In this approach, points are evenly retained from the original dataset according to a fixed interval to achieve a similar reduction ratio as the proposed method. Table 2 presents the comparison results in terms of data size, computational cost, and measurement accuracy. As shown in Table 2, both the proposed method and uniform subsampling achieve a similar level of data reduction (approximately 66%). The uniform subsampling method exhibits slightly lower computational cost due to its simplicity. However, it leads to a significant increase in depth estimation error and a noticeable decrease in peak detection accuracy, as important geometric features may be randomly discarded. In contrast, the proposed method selectively preserves points with high geometric significance based on angle and chord deviation constraints. As a result, it achieves a substantially lower depth estimation error (reduced by approximately 60%) and significantly higher feature extraction accuracy compared to uniform subsampling. These results demonstrate that the proposed preprocessing strategy provides a better trade-off between computational efficiency and measurement accuracy, validating its necessity for reliable industrial inspection.

## 6. Conclusions

This study focuses on the measurement and evaluation of corrugated plate depths in plate heat exchangers and proposes a rapid and accurate measurement approach based on line laser sensing. The key findings are summarized as follows:

(1) Development of measurement methodology:

A non-contact measurement system integrating a line laser sensor, encoder, and linear guide rail was developed for continuous surface profile acquisition. The proposed system enables full-field, high-resolution inspection of corrugated plates, facilitating comprehensive characterization of geometric features over the entire surface. Compared with conventional coordinate measuring machine (CMM)-based point-wise inspection, it offers a more efficient and scalable solution for full-coverage dimensional measurement.

(2) Data reduction strategy and efficiency improvement:

To address the large data volume and low computational efficiency associated with line laser acquisition, a data condensation technique based on an integrated angle–chord height error criterion was proposed. The method reduces the number of data points from 3200 to 1086 (approximately 66%) while preserving essential curvilinear features. This significantly lowers the computational burden of subsequent analysis and improves overall processing efficiency.

(3) Feature identification and depth calculation:

A two-tiered method for peak and valley detection was developed. The first stage identifies local extrema in the reduced 2D point cloud to estimate feature positions, while the second stage applies local B-spline fitting combined with a bisection method to accurately determine the true extremum points. By mitigating errors caused by discrete sampling misalignment, this approach improves the accuracy and robustness of feature extraction. Validation against CMM measurements shows that the maximum deviation is within 17%, confirming the reliability of the proposed method.

Compared with uniform subsampling, the proposed preprocessing strategy improves depth estimation accuracy by approximately 60% while achieving a comparable level of data reduction, demonstrating its effectiveness in feature-preserving data compression. Overall, the proposed methodology ensures measurement accuracy while significantly enhancing both measurement and computational efficiency, providing a reliable and efficient solution for rapid depth evaluation of corrugated plates in plate heat exchangers.

## Figures and Tables

**Table 1:** Nominal parameters of line laser-vision sensor (LJ-X8400).

Parameter	Reference Distance	Z-Axis Height	X-Axis Width	Z-Axis Repeatability	X-Axis Repeatability
Specifications	380 mm	±60 mm	180 mm	5 μm	10 μm

**Table 2:** Comparative experiment with Uniform Subsampling Method.

Method	Data Points	Reduction Ratio	Runtime (ms)	Depth Error (mm)	Peak Detection Accuracy (%)
Raw data	3200	0%	42	0.021	100
Uniform subsampling	1080	66.3%	15	0.087	82.5
Proposed method	1086	66.1%	18	0.034	96.8

## Data Availability

The original contributions presented in this study are included in the article. Further inquiries can be directed to the corresponding author.
